# Explaining the difference in prognosis between screen-detected and symptomatic breast cancers

**DOI:** 10.1038/bjc.2011.144

**Published:** 2011-05-03

**Authors:** P C Allgood, S W Duffy, O Kearins, E O'Sullivan, N Tappenden, M G Wallis, G Lawrence

**Affiliations:** 1 CR-UK Centre for Epidemiology, Mathematics and Statistics, Wolfson Institute for Preventive Medicine, Charterhouse Square, London WC1M 6BQ, UK; 2 West Midlands Cancer Intelligence Unit, The University of Birmingham, Birmingham B15 2TT, UK; 3 Cambridge Breast Unit, Addenbrookes Hospital, Cambridge, UK

**Keywords:** breast cancer, screening, prognosis, survival, lead time bias

## Abstract

**Background:**

We analysed 10-year survival data in 19 411 women aged 50–64 years diagnosed with invasive breast cancer in the West Midlands region of the United Kingdom. The aim was to estimate the survival advantage seen in cases that were screen detected compared with those diagnosed symptomatically and attribute this to shifts in prognostic variables or survival differences specific to prognostic categories.

**Methods:**

We studied tumour size, histological grade and the Nottingham Prognostic Index in very narrow categories and investigated the distribution of these prognostic factors within screen-detected and symptomatic tumours. We also adjusted for lead time bias.

**Results:**

The unadjusted 10-year breast cancer survival in screen-detected cases was 85.5% and in symptomatic cases 62.8% after adjustment for lead time bias, survival in the screen-detected cases was 79.3%. Within narrow categories of prognostic variables, survival differences were small, indicating that the majority of the survival advantage of screen detection is due to differences in the distributions of size and node status.

**Conclusion:**

Our results suggested that a combination of lead time with size and node status in 10 categories explained almost all (97%) of the survival advantage. Only a small proportion remained to be explained by biological differences, manifested as length bias or overdiagnosis.

Breast cancer screening with mammography is known to reduce mortality from the disease (Smith *et al*, 2004) and although there is some dissent (Gøtzsche *et al*, 2009), the majority opinion is that mammographic screening is effective (Vainio, 2002). The major mechanism of this mortality reduction is the diagnosis of disease at an early stage, while it is likely to be successfully treatable (Tabár *et al*, 1985; Smith *et al*, 2004).

In recent years, there has been interest in the extent to which screen-detected breast cancer differs from symptomatic disease in biological terms (Collett *et al*, 2005; Wishart *et al*, 2008). Survival studies have indicated that the majority of the survival benefit can be attributed to smaller size and a lesser rate of node involvement at presentation (Wishart *et al*, 2008). Biological variables such as HER-2 status apparently account for <10% of the difference in prognosis between screen-detected and symptomatic cancers (Dawson *et al*, 2009). Around 30% of the difference remains to be explained (Wishart *et al*, 2008; Dawson *et al*, 2009).

It is also of interest to study survival differences in narrow prognostic categories, to ascertain whether the difference can be better explained by more minute categorisation of factors such as tumour size, and whether the survival advantage of screen-detected tumours is more marked in higher risk or lower risk tumours. It is also desirable to take lead time into account in explaining survival differences.

In this paper, we investigate the proportion of the survival difference between screen-detected and symptomatic tumours that can be explained by tumour size, a combination of tumour size and node status, histological grade and the Nottingham Prognostic Index (NPI), which takes into account all three prognostic factors. In addition, we estimate the difference that can be explained by lead time, the additional observation time added to the survival as a result of early detection by screening.

We also use a method, described by Bashir and Estève (2000), for partitioning the variation in survival between the two modes of breast cancer detection (screening or symptomatic) with respect to (1) the distribution of prognostic factors by detection mode and (2) differences in survival specific to prognostic factor status in narrow categories. In this study, we used 19 411 invasive breast tumours diagnosed in women aged 50–64 years recorded by the West Midlands Cancer Intelligence Unit. The size of the remaining survival differences, between screen-detected and symptomatic tumours after taking into account lead time and the difference in pathological prognostic factors illustrates the scope of survival differences attributable to length bias and overdiagnosis. Length bias in the context of screening is the tendency of screening to detect preferentially more slow-growing tumours, which therefore have better prognosis. Overdiagnosis is the extreme form of length bias whereby screening detects some tumours, which would never have been diagnosed in the host's lifetime had the screening not taken place.

## Materials and methods

In collaboration with the NHS Breast Screening Programme, the West Midlands Cancer Intelligence Unit aims to determine the screening histories of all women diagnosed with breast cancer in the West Midlands, UK. Screening histories for 19 411 women aged between 50 and 64 years with invasive breast tumours diagnosed between 1988 and 2004; 11 674 (60.1%) diagnosed symptomatically and 7737 (39.9%) screen detected are included in this study. We studied the survival difference between symptomatic and screen-detected tumours in relation to tumour size, grade, nodal status and the NPI. The latter is a validated prognostic tool based on tumour size, grade and lymph node status (Todd *et al*, 1987). It is frequently categorised into five prognostic groups (Lee and Ellis, 2008): excellent (NPI<2.41), good (2.41⩽NPI<3.41), moderate 1 (3.41⩽NPI<4.41), moderate 2 (4.41⩽NPI<5.41) and poor (NPI⩾5.41). Note that the number of cases vary among analyses, due to different numbers with missing data on size, node status and grade. We also considered socioeconomic status as measured by the area-based Townsend score.

Categorical variables were compared between symptomatic and screen-detected tumours using the *χ*^2^-test, and continuous variables using the Wilcoxon test (Wilcoxon, 1945). For survival analysis, we first examined the difference in 10-year Kaplan–Meier survival (Kaplan and Meier, 1958) in five size categories between symptomatic and screen-detected tumours. We then estimated the expected overall survival for the symptomatic cases if they had had the same size distribution as the screen-detected cases, using the method of Bashir and Estève (2000). This yielded an estimate of the proportion of the survival difference attributable to the more favourable size distribution of screen-detected cancers, the complementary proportion attributable to size-specific survival differences between the two detection modes.

The analysis was performed with and without adjustment for lead time. We repeated this analysis for size categorised into 10 classes, for a combination of tumour size and node status, for histological grade and for the NPI, divided into 10 prognostic groups. We adjusted for lead time bias using the method of Duffy *et al* (2008), who estimated the additional time of observation, due to screening lead time, between diagnosis and either death or censoring for each screen-detected case. They showed that for a subject who dies of breast cancer at time *t*, the additional time is on average 
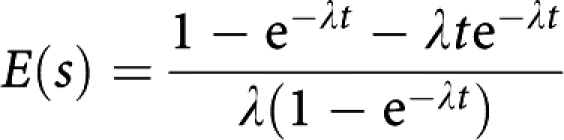


For a subject censored at time *t*, the average additional time is 
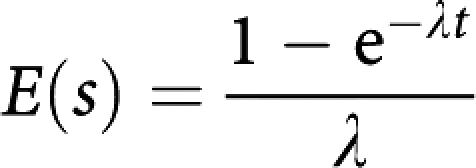
 where *λ* is the rate of transition from asymptomatic to symptomatic disease, and is the reciprocal of the average asymptomatic screen-detectable period. We calculated *E*(*s*) for every screen-detected case, and subtracted this from their survival time. We estimated *λ* as 0.26 from the largest of the breast cancer screening trials (Tabár *et al*, 2000). This corresponds to an average asymptomatic screen-detectable period of 3.9 years.

With the correction for lead time, the proportion of the survival difference accounted for by pathological prognostic factors such as size can be considered the residual proportion attributable to size etc, after removal of the lead time effect. The difference remaining to be accounted for is attributable to unobserved factors, and to length bias or overdiagnosis.

The above analysis was complemented by Cox proportional hazards regression (Clayton and Hills, 1993), estimating the relative hazard for screen-detected cancers unadjusted and adjusted for pathological factors and lead time. In addition, the Freedman statistic for the proportion of the survival difference accounted for by the various adjustment factors was calculated (Freedman *et al*, 1992).

## Results

Patient and tumour characteristics are shown for screen-detected and symptomatic cases in Table 1. All variables showed significant differences between the two detection modes. The symptomatic cases were slightly but significantly younger and slightly but significantly more deprived, had larger tumours, had a greater proportion of tumours with positive nodes and had tumours with a more severe grade. Consequently, women with symptomatic tumours had a poorer prognosis than women with cancers detected by screening.

Table 2 shows invasive breast tumours categorised into five size groups for symptomatic and screen-detected tumours and their 10-year survival rates. The unadjusted 10-year survival for women with screen-detected tumours compared to women with symptomatic tumours was better in all size groups and overall. This was most marked in the 21–50 mm size groups, and the adjustment for lead time had the strongest attenuating effect in these groups. Note that the overall survival difference is greater than observed within specific size categories. This indicates that a substantial part of the survival benefit of screen detection is due not to size-specific differences but to shifts in tumour size associated with screen detection. This phenomenon was also observed in subsequent analyses described below. Overall, the absolute survival advantage for women with screen-detected tumours was 85.9–65.3=20.6%.

The expected overall survival in the symptomatic cases if they had had the same size distribution as the screen detected was calculated as 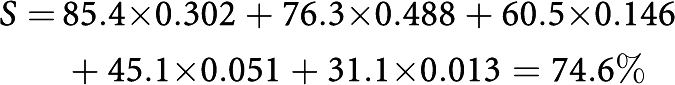


The proportion of the survival difference explained by the different size distributions was therefore 
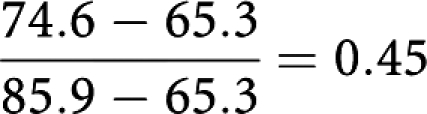


That is, 45% of the survival difference between screen-detected and symptomatic cases can be attributed to the more favourable size distribution (using these five size categories) in the screen-detected tumours, and 55% to differences in size-specific survival. The overall 10-year survival of the screen-detected tumours adjusted for lead time was 79.8%. This suggests that 30% of the difference is due to lead time. The proportion of the remaining survival difference attributable to the differing size distributions was 
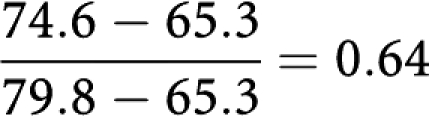


That is, 64% of the difference in survival after adjustment for lead time is attributable to the better size distribution of screen-detected cases.

Survival differences were markedly changed when the tumours were divided by size and node status simultaneously (Table 3). For node-negative tumours, the greatest survival advantage for screen-detected cases was in the 31–50 mm size group for both unadjusted and adjusted figures. The smallest difference was seen in the smallest tumours where indeed a slight survival advantage was observed for women with symptomatic tumours after adjustment for lead time. For node-positive tumours, the greatest survival advantage was seen in women with the smallest tumours using either unadjusted or adjusted survival figures.

The expected survival in the symptomatic tumours if they had had the same size and node status distribution as the screen-detected cases was 77.0%. Unadjusted for lead time, the overall survival of the screen-detected tumours was 85.0%. Thus, before adjusting for lead time, 60% of the survival advantage of screen-detected tumours was attributable to the difference between the joint distributions of tumour size and node status. After adjustment for lead time, the survival difference between screen-detected and symptomatic tumours was 12.5%, and the difference between the survival of screen-detected cases and that expected in the symptomatic if they had had the same size/node status distribution as the screen detected was 77.4–77.0=0.4%. Thus, almost all (97%) of the remaining survival difference after adjusting for lead time was attributable to the difference between screen-detected and symptomatic tumours in terms of size and node status.

Table 4 shows invasive breast tumours categorised into histological grade for symptomatic and screen-detected tumours and their 10-year survival rates. The unadjusted and adjusted 10-year survivals for the screen-detected cases compared with the symptomatic cases was better for all grades and overall although less after adjusting for lead time. Overall, the absolute survival advantage for women with screen-detected tumours was 85.0–64.9=20.1% unadjusted. The expected overall survival in the symptomatic cases if they had had the same size distribution as the screen detected was 71.6%. The proportion of the survival difference explained by the different size distributions was 34%, so 66% was due to the difference in grade-specific survival. The overall 10-year survival of the screen-detected tumours adjusted for lead time was 77.4%. This suggests that 37.6% of the difference is due to lead time. The proportion of the remaining survival difference attributable to the differing grade distributions was 0.54, that is, 54% of the difference in survival after adjustment for lead time is attributable to the better grade distribution of screen-detected cases. The greatest survival advantage was seen in women with grade 2 tumours both before and after adjustment for lead time and the smallest difference was seen for women with grade 1 tumours.

Since size, node status and grade are correlated, the attributable percentages are non-exclusive and cannot be combined additively. Table 5 shows 10-year survival for symptomatic and screen-detected cases when tumours were divided into 10 NPI categories. Total survival for the symptomatic tumours was 66.1%, and for the screen-detected tumours, 84.7% unadjusted, and 75.5% after adjustment for lead time. There was a screen-detected survival advantage for all prognostic groups when using the unadjusted survival figures except for women in the 4.21<4.38 group where a small survival advantage for women with symptomatic tumours was seen. When using lead time adjusted survival figures, there was an even larger survival advantage seen for women with symptomatic tumours in this prognostic group. The expected survival for symptomatic tumours if they had had the same NPI distribution as the screen-detected cases was 79.7%. Thus, the NPI distribution accounted for 73% of the survival difference without adjustment for lead time and entirely accounted for the difference after lead time adjustment.

For some of the categories, the survival in the screen-detected tumours is poorer after lead time adjustment. This may be due to the fact that much of the lead time is highly correlated with the prognostic factors making up the NPI and therefore within very minute categories of NPI there is little residual lead time, and therefore the correction may be an overadjustment.

Table 6 shows the relative hazard for screen-detected *vs* symptomatic cancers, unadjusted and adjusted for prognostic factors, and uncorrected and corrected for lead time. The Freedman statistics indicate that size and node status account for 46% of the survival difference, and that correction for lead time, size and node status account for 90% of the difference. The NPI accounts for 67% of the difference, but together with the correction for lead time, it accounts for 100% of the difference. These results are consistent with those of the Bashir and Estève (2000) method. The lead time corrected and NPI adjusted results again suggest an overcorrection.

## Discussion

We analysed the 10-year survival data of 19 411 women aged 50–64 years diagnosed with invasive breast cancers in the West Midlands region of the United Kingdom. The availability of the very large tumour series with detailed screening history made it possible to divide the cancers into very narrow prognostic bands. Our results found a strong survival advantage for women with screen-detected tumours as seen in many studies comparing screen-detected and symptomatic breast cancers (Wishart *et al*, 2008; Dawson *et al*, 2009; Lawrence *et al*, 2009). The survival advantage was partly explained by the more favourable distribution of tumour size in narrow prognostic categories. When screen-detected tumour survival was additionally adjusted for lead time, the survival advantage was still evident, but smaller. When the tumours were classified into 10 categories by size and node status, the survival difference was almost entirely accounted for by a combination of lead time and the more favourable size and node status of the screen-detected cancers, with a remaining absolute survival difference of <1%.

A strong survival advantage was also seen for women with screen-detected tumours when adjusted for histological grade, which was again, attenuated when adjusted for lead time. Simultaneously adjusting for lead time and NPI, which incorporates tumour size, node status and histological grade, the survival difference between screen-detected and symptomatic tumours was entirely accounted for. However, one might argue that histological grade in many cases is an innate feature of tumour biology rather than a time-progressive attribute of the tumour, so the size–node status adjustment might be more appropriate.

The lead time adjustment is rigorous and based on empirical estimation of the average preclinical screen-detectable period from a large randomised trial, estimating the average sojourn time as 3.9 years (Tabar *et al*, 2000). This gave an average additional observation due to lead time of 3 years in the screen-detected cases in our data. The method depends also on the observed survival time, so that the lead time correction is on average smaller for poor prognosis tumours than for tumours with favourable prognostic attributes. This makes overcorrection unlikely, although there may be some overcorrection within prognostic categories defined partially by non-progressive features. This may be the case for the NPI results, since at the very least for some tumours the grade is an innate rather than a progressive characteristic of the tumour. There is a wide range of sojourn time estimates in the literature, and a shorter mean sojourn time would give a smaller proportion of the survival difference accountable for by lead time. However, the estimated mean sojourn times vary by age and in this age group, 50–64 years, they are mostly close to our estimate of 3.9 years (Paci and Duffy, 1991; Tabar *et al*, 2000; Weedon-Fekjaer *et al*, 2005).

The conclusion up to now has been that the portion of the survival advantage of screen-detected cancers that could not be attributed to the prognostic factors size and node status (and possibly grade) must be attributable to unobserved biological covariates (Collett *et al*, 2005; Wishart *et al*, 2008). Our results suggest that a combination of lead time with size and node status in 10 categories explains almost all of the survival advantage. This does not invalidate the hypothesis of further unobserved biological differences, since biological tumour features will almost certainly affect tumour progression rates and therefore lead time. It does, however, suggest that only a small proportion of the survival advantage of screen-detected cancers remains to be explained by biological differences between screen-detected and symptomatic tumours.

Such biological differences are likely to give rise to length bias, the tendency of screening to detect the more slow-growing tumours. The extreme form of length bias is overdiagnosis, the detection by screening of cancers that would never have been diagnosed in the host's lifetime if screening had not taken place. Estimates of overdiagnosis vary considerably (Biesheuvel *et al*, 2007). The results here do not formally estimate the overdiagnosis rate, but the small amount of the survival benefit that remains unattributed after correction for lead time and adjustment for tumour size and node status would only require a small degree of overdiagnosis (between 3% and 10%) to account for it.

In addition to the survival difference conferred by different distributions of prognostic factors, there were notable differences in survival within prognostic categories. Broadly, substantially better survival was observed with screen detection for node-negative tumours of size 21–50 mm and node-positive tumours of size ⩽30 mm. These were partly but not entirely explained by lead time.

In conclusion, in this large tumour series, the better survival of screen-detected breast cancers was almost entirely explained by a combination of lead time and the improved size and node status of screen-detected tumours.

## Figures and Tables

**Table 1:** Patient characteristics by mode of detection

**Mode of detection**	**Screen detected**	**Symptomatic**	**Test for difference**
Total numbers	7737	11 674	
			
*Townsend deprivation quintile,* N *(%)*			
Least deprived 1	2067 (26.7)	2975 (25.5)	*χ*^2^ for trend=12.32, *P*=0.002
2	1745 (22.6)	2553 (21.7)	
3	1522 (19.7)	2315 (19.9)	
4	1427 (18.5)	2210 (19.0)	
Most deprived 5	969 (12.5)	1621 (13.9)	
Unknown	7	0	
			
*Age at diagnosis*			
Mean (s.d.)	57.2 (4.3)	56.8 (4.4)	Wilcoxon rank sum *Z*=−5.02, *P*<0.001
Median (IQR)	57 (53–61)	57 (53–61)	
			
*Size of tumour*			
Mean (s.d.)	16.5 (10.9)	26.1 (18.8)	Wilcoxon rank sum *Z*=46.65, *P*<0.0001
Median (IQR)	15 (10–20)	20 (15–30)	
*N*, unknown	690	2447	
			
*Nodal status*			
Positive	1722 (27.0)	4395 (48.1)	Pearson *χ*^2^ (1), *P*<0.001
Negative	4648 (73.0)	4735 (51.9)	
Not examined/ unknown	1367	2544	
			
*Histological grade*			
1	2045 (31.9)	1099 (12.6)	*χ*^2^ (3)=1327, *P*<0.001
2	3038 (47.4)	3719 (42.7)	
3	1327 (20.7)	3898 (44.7)	
Unknown	1327	2958	
			
*NPI*			
Excellent	1320 (24.7)	521 (7.7)	*χ*^2^ for trend=1600, *P*<0.001
Good	1775 (33.3)	1239 (18.4)	
Moderate 1	1264 (23.7)	1744 (25.8)	
Moderate 2	598 (11.2)	1657 (24.6)	
Poor	381 (7.1)	1589 (23.5)	
Unknown	2399	4924	

Abbreviations: IQR=interquartile range; NPI=Nottingham Prognostic Index.

**Table 2:** 10-year survival for women aged 50–64 years with symptomatic and screen-detected invasive breast tumours by size of tumour in five categories

			**Screen detected**				
	**Symptomatic**			**10-year survival (%)**			
**Size (mm)**	***N* (%)**	**10-year survival (%)**	***N* (%)**	**Unadjusted for lead time**	**Difference from symptomatic**	**Adjusted for lead time**	**Difference from symptomatic**
<11	971 (10.5)	85.4	2125 (30.2)	94.3	8.9	90.9	5.5
11<21	3688 (40.0)	76.3	3442 (48.8)	87.2	10.9	80.4	4.1
21<31	2549 (27.6)	60.5	1029 (14.6)	77.2	16.7	69.3	8.8
31<51	1426 (15.5)	45.1	358 (5.1)	63.3	18.2	53.7	8.6
51+	593 (6.4)	31.1	93 (1.3)	41.3	10.2	38.8	7.7
Overall	9227 (100)	65.3	7047 (100)	85.9	20.6	79.8	14.5

**Table 3:** 10-year survival for women aged 50–64 years with symptomatic and screen-detected invasive breast tumours by a combination of tumour size and nodal status

			**Screen detected**				
	**Symptomatic**			**10-year survival (%)**			
**Size (mm) and node status**	***N* (%)**	**10-year survival (%)**	***N* (%)**	**Unadjusted for lead time**	**Difference from symptomatic**	**Adjusted for lead time**	**Difference from symptomatic**
<11, Negative	564 (7.2)	93.2	1505 (25.3)	95.1	1.9	91.5	−1.7
11–20, Negative	2008 (25.7)	84.1	2178 (36.6)	91.9	7.8	84.5	0.4
21–30, Negative	1073 (13.7)	74.8	521 (8.8)	85.0	10.2	76.3	1.5
31–50, Negative	433 (5.5)	59.8	135 (2.3)	77.7	17.9	68.8	9.0
51+, Negative	116 (1.5)	46.3	28 (0.5)	56.2	9.9	54.1	7.8
<11, Positive	167 (2.1)	62.4	162 (2.7)	85.7	23.3	80.4	18.0
11<21, Positive	1089 (13.9)	62.7	769 (12.9)	73.9	11.2	62.9	0.2
21<31, Positive	1135 (14.5)	47.6	403 (6.8)	64.4	16.8	56.3	8.7
31<51, Positive	835 (10.7)	36.5	196 (3.3)	51.4	14.9	38.8	2.3
51+, Positive	393 (5.0)	26.1	55 (0.9)	32.7	6.6	29.6	3.5
Overall	7813 (100.0)	64.9	5952 (100.0)	85.0	20.1	77.4	12.5

**Table 4:** 10-year survival for women aged 50–64 years with symptomatic and screen-detected invasive breast tumours by histological grade of tumour

			**Screen detected**				
	**Symptomatic**			**10-year survival (%)**			
**Grade**	***N* (%)**	**10-year survival (%)**	***N* (%)**	**Unadjusted for lead time**	**Difference from symptomatic**	**Adjusted for lead time**	**Difference from symptomatic**
1	1099 (12.6)	87.3	2045 (31.9)	95.1	7.8	88.9	1.6
2	3719 (42.7)	68.1	3038 (47.4)	85.6	17.5	77.8	9.7
3	3898 (44.7)	55.4	1327 (20.7)	68.5	13.1	60.5	5.1
Overall	8716 (100)	64.8	6410 (100)	85.0	20.2	77.4	12.6

**Table 5:** 10-year survival by mode of detection for women aged 50–64 years with invasive breast tumours in 10 NPI categories

			**Screen detected**				
	**Symptomatic**			**10-year survival**			
**NPI**	***N* (%)**	**10-year survival (%)**	***N* (%)**	**Unadjusted for lead time**	**Difference from symptomatic**	**Adjusted for lead time**	**Difference from symptomatic**
<2.3	299 (4.4)	94.3	997 (18.7)	96.4	2.1	94.6	0.3
2.30<3.17	415 (6.2)	88.1	706 (13.2)	96.0	7.9	87.5	−0.6
3.17<3.30	392 (5.8)	88.7	766 (14.4)	95.0	6.3	88.9	0.2
3.30<3.41	654 (9.7)	85.7	626 (11.7)	91.1	5.4	82.5	−3.2
3.41<4.21	749 (11.1)	74.9	576 (10.8)	85.5	10.6	75.9	1.0
4.21<4.38	654 (9.7)	80.9	524 (9.8)	78.6	−2.3	62.8	−18.1
4.38<4.51	808 (12.0)	71.2	352 (6.6)	74.6	3.4	69.6	−1.6
4.51<5.28	826 (12.2)	59.0	257 (4.8)	70.4	11.4	60.4	1.4
5.28<5.70	961 (14.2)	48.3	325 (6.1)	55.0	6.7	38.9	−9.4
⩾5.70	992 (14.7)	28.6	209 (3.9)	35.1	6.5	23.3	−5.3
Total	6750 (100)	66.1	5338 (100)	84.7	18.6	75.5	9.4

Abbreviation: NPI=Nottingham Prognostic Index.

**Table 6:** Cox regression results – relative hazards for screen-detected *vs* symptomatic tumours, the effect of adjustment for prognostic factors and correction for lead time, and the Freedman (199 percentage of the survival advantage of screen-detected tumours accounted for by adjustment and correction)

**Correction for lead time**	**Adjustment**	**Relative hazard (95% CI)**	**Freedman percentage**
No	None	0.34 (0.31–0.36)	—
	Size and node status	0.56 (0.50–0.61)	46
	NPI	0.70 (0.63–0.78)	67
Yes	None	0.48 (0.45–0.52)	32
	Size and node status	0.90 (0.82–0.99)	90
	NPI	1.20 (1.09–1.33)	100

Abbreviations: CI=confidence interval; NPI=Nottingham Prognostic Index.

## References

[bib1] Bashir SA, Estève J (2000) Analysing the difference due to risk and demographic factors for incidence or mortality. Int J Epidemiol 29: 878–884 11034972 10.1093/ije/29.5.878

[bib2] Biesheuvel C, Barratt A, Howard K, Houssami N, Irwig L (2007) Effects of study methods and biases on estimates of invasive breast cancer overdetection with mammography screening: a systematic review. Lancet Oncol 8: 1129–1138 18054882 10.1016/S1470-2045(07)70380-7

[bib3] Clayton D, Hills M (1993) Statistical Models in Epidemiology. Oxford University Press: Oxford

[bib4] Collett K, Stefansson IM, Eide J, Braaten A, Wang H, Eide GE, Thoresen SO, Foulkes WD, Akslen LA (2005) A basal phenotype is more frequent in interval breast cancers compared with screen-detected tumours. Cancer Epidemiol Biomarkers Prev 14: 1108–1112 15894660 10.1158/1055-9965.EPI-04-0394

[bib5] Dawson SJ, Duffy SW, Blows FM, Driver KE, Provenzano E, LeQuesne J, Greenberg DC, Pharoah P, Caldas C, Wishart GC (2009) Molecular characteristics of screen-detected vs symptomatic breast cancers and their impact on survival. Br J Cancer 101: 1338–1344 19773756 10.1038/sj.bjc.6605317 PMC2768460

[bib6] Duffy SW, Nagtegaal ID, Wallis M, Cafferty FH, Houssami N, Warwick J, Allgood PC, Kearins O, Tappenden N, O’Sullivan E, Lawrence G (2008) Correcting for lead time and length bias in estimating the effect of screen detection on cancer survival. Am J Epidemiol 168: 98–104 18504245 10.1093/aje/kwn120

[bib7] Freedman LS, Graubard BI, Schatzkin A (1992) Statistical validation of intermediate endpoints for chronic diseases. Stat Med 11: 167–178 1579756 10.1002/sim.4780110204

[bib8] Gøtzsche PC, Hartling OJ, Nielsen M, Brodersen J, Jørgensen KJ (2009) Breast screening, the facts – or maybe not. BMJ 338: 446–448 10.1136/bmj.b86 19174442

[bib9] Kaplan EL, Meier P (1958) Non parametric estimation from incomplete observations. J Am Stat Assoc 53: 457–481

[bib10] Lawrence G, O’Sullivan E, Kearins O, Tappenden N, Martin K, Wallis M (2009) Screening histories of invasive breast cancers diagnosed 1989–2006 in the West Midlands, UK: variation with time and impact on 10-year survival. J Med Screen 16: 186–192 20054093 10.1258/jms.2009.009040

[bib11] Lee AHS, Ellis IO (2008) The Nottingham Prognostic Index for invasive carcinoma of the breast. Pathol Oncol Res 14: 113–115 18543079 10.1007/s12253-008-9067-3

[bib12] Paci E, Duffy SW (1991) Modelling the analysis of breast cancer screening programme: sensitivity, lead time and predictive value in the Florence District Programme (1975–1986). Int J Epidemiol 20: 852–858 1800422 10.1093/ije/20.4.852

[bib13] Smith RA, Duffy SW, Gabe R, Tabar L, Yen AMF, Chen HHT (2004) The randomized trials of breast cancer screening: what have we learned? Radiol Clin N Am 42: 793–806 15337416 10.1016/j.rcl.2004.06.014

[bib14] Tabár L, Gad A, Holmberg LH, Ljungquist U, Fagerberg CJG, Baldetor L, Gröntoft O, Lundström B, Månson JC, Eklund G, Day NE, Pettersson F, Kopparberg County Project Group, Östergötland County Project Group (1985) Reduction in mortality from breast cancer after mass screening with mammography. Lancet 325: 829–832 10.1016/s0140-6736(85)92204-4 2858707

[bib15] Tabár L, Vitak B, Chen HH, Duffy SW, Smith RA (2000) The Swedish Two-County Trial twenty years later: updated mortality results and new insights from long term follow-up. Radiol Clin N Am 38: 625–651 10943268 10.1016/s0033-8389(05)70191-3

[bib16] Todd JH, Doyle C, Williams MR, Elston CW, Hinton CP, Blamey RW, Haybittle JL, Nicholson RI, Griffiths K (1987) Confirmation of a prognostic index in primary breast cancer. Br J Cancer 56: 489–492 3689666 10.1038/bjc.1987.230 PMC2001834

[bib17] Vainio H (2002) IARC Handbooks of Cancer Prevention: Breast Cancer Screening. IARC Press: Lyon

[bib18] Weedon-Fekjaer H, LIndqvist BH, Vatten LJ, Aalen O, Tretli S (2005) Estimating mean sojourn time and screening test sensitivity in breast cancer mammography screening: new results. J Med Screen 12: 172–178 16417693 10.1258/096914105775220732

[bib19] Wilcoxon F (1945) Individual comparisons by ranking methods. Biometrics 1: 80–83 18903631

[bib20] Wishart GC, Greenberg DC, Britton PD, Chou P, Brown CH, Purushotham AD, Duffy SW (2008) Screen-detected vs symptomatic breast cancer: is improved survival due to stage migration alone? Br J Cancer 98: 1741–1744 18506175 10.1038/sj.bjc.6604368 PMC2410118

